# Integrated proteome sequencing, bulk RNA sequencing and single-cell RNA sequencing to identify potential biomarkers in different grades of intervertebral disc degeneration

**DOI:** 10.3389/fcell.2023.1136777

**Published:** 2023-03-16

**Authors:** Xiao Yang, Yang Lu, Hang Zhou, Hai-Tao Jiang, Lei Chu

**Affiliations:** Department of Orthopaedics, The Second Affiliated Hospital of Chongqing Medical University, Chongqing, China

**Keywords:** bioinformatics, machine learning, bulk RNA sequencing, intervertebral disc degeneration, single-cell RNA sequencing, proteome sequencing

## Abstract

Low back pain (LBP) is a prevalent health problem worldwide that affects over 80% of adults during their lifetime. Intervertebral disc degeneration (IDD) is a well-recognized leading cause of LBP. IDD is classified into five grades according to the Pfirrmann classification system. The purpose of this study was to identify potential biomarkers in different IDD grades through an integrated analysis of proteome sequencing (PRO-seq), bulk RNA sequencing (bRNA-seq) and single-cell RNA sequencing (scRNA-seq) data. Eight cases of grade I-IV IDD were obtained. Grades I and II were considered non-degenerative discs (relatively normal), whereas grades III and IV were considered degenerative discs. PRO-seq analysis was performed to identify differentially expressed proteins (DEPs) in various IDD grades. Variation analysis was performed on bRNA-seq data to differentiate expressed genes (DEGs) in normal and degenerated discs. In addition, scRNA-seq was performed to validate DEGs in degenerated and non-degenerated nucleus pulposus (NP). Machine learning (ML) algorithms were used to screen hub genes. The receiver operating characteristic (ROC) curve was used to validate the efficiency of the screened hub genes to predict IDD. Gene Ontology (GO) and Kyoto Encyclopedia of Genes and Genomes (KEGG) analyses were performed to analyze function enrichment and signaling pathways. Protein-protein interaction (PPI) network was used to prioritize disease-related proteins. SERPINA1, ORM2, FGG and COL1A1 were identified through PRO-seq as the hub proteins involved in regulating IDD. ML algorithms selected ten hub genes, including IBSP, COL6A2, MMP2, SERPINA1, ACAN, FBLN7, LAMB2, TTLL7, COL9A3, and THBS4 in bRNA-seq. Since serine protease inhibitor clade A member 1 (SERPINA1) was the only common gene, its accuracy in degenerated and non-degenerated NP cells was validated using scRNA-seq. Then, the rat degeneration model of caudal vertebra was established. The expression of SERPINA1 and ORM2 was detected using immunohistochemical staining of human and rat intervertebral discs. The results showed that SERPINA1 was poorly expressed in the degenerative group. We further explored the potential function of SERPINA1 by Gene Set Enrichment Analysis (GSEA) and cell-cell communication. Therefore, SERPINA1 can be used as a biomarker to regulate or predict the progress of disc degeneration.

## 1 Introduction

Low back pain (LBP) is a prevalent health problem worldwide that affects over 80% of adults during their lifetime (Cazzanelli and Wuertz-Kozak, 2020; Borenstein and Balagué, 2021). LBP has been the leading cause of disability in recent decades, especially in low- and middle-income countries (Hartvigsen et al., 2018). Intervertebral disc degeneration (IDD) is a major cause of LBP and the pathological basis of spinal diseases, such as spinal stenosis, radiculopathy, spur formation, lumbar disc herniation and lumbar vertebral instability (Wang et al., 2020). These diseases directly affect patients’ quality of life, and the treatment costs may be enormous for impoverished households (Hoy et al., 2012). An intervertebral disc (IVD), a fibrocartilage structure with great elasticity and tenacity, is located between two adjacent vertebrae. IVD is composed of the annulus fibrosus (AF), the inner nucleus pulposus (NP), and the cartilage endplate (CEP) on the superior and inferior sides of the disk (Yang et al., 2020). IVD can protect vertebral bones, the brain, and the spinal cord from internal and external shock and stress, as well as extend the range of motion of the spine (Hickman et al., 2022). In addition, IVD has a unique microenvironment characterized by mechanical loading, hypertonicity, hypoxia, nutrient deficiency, high acidity, and low cell density (Sakai and Grad, 2015). In a state of disequilibrium, a series of pathological changes occur in the IVD. NP cells are the inner core of the IVD. Therefore, during degeneration, several changes gradually occur in these cells, including cell reduction, increased apoptosis, decreased extracellular matrix (ECM) secretion, and increased expression of matrix degradation proteins (Wang et al., 2020). The degeneration of IVD is accompanied by the loss of proteoglycans and collagen fibers, eventually leading to a decrease in IVD height (Sakai and Grad, 2015). Magnetic resonance imaging is a highly sensitive method to detect IDD. IVD degeneration is classified into five grades according to the Pfirrmann classification system (Pfirrmann et al., 2001). With the rapid development of proteome sequencing (PRO-seq), bulk RNA sequencing (bRNA-seq) and single-cell RNA sequencing (scRNA-seq) technologies, multi-omics analysis has become a focal point of current research. Numerous studies have shown that proteins play a significant role in IDD (Cheng et al., 2018; Che et al., 2020; Chao-Yang et al., 2021). Messenger RNA is a type of RNA molecule that can transfer genetic information from DNA to ribosomes and serves as a template for protein synthesis (Morris et al., 2021). Studies have shown that several genes play an important role in the degenerative process of IDD, such as aggrecan, interleukin, and matrix metalloproteases (Roughley et al., 2006; Wang et al., 2015; Wang et al., 2020). Previous bioinformatics studies identified candidate regulatory targets of IDD through RNA-seq analysis (Li et al., 2021). The scRNA-seq analysis identified a new group of chondrocytes in IDD and also proved the correlation between ferroptosis and IDD (Zhang et al., 2021b). Therefore, screening potential biomarkers of IDD *via* multi-omics analysis is of great clinical significance. Herein, we integrated three levels of omics analysis, including PRO-seq, bRNA-seq, and scRNA-seq, and performed tissue experiments using human and mice IVD tissues. IVD tissue samples with different degrees of degeneration (grades I-IV) were collected for PRO-seq analysis of the potential hub proteins in IDD, which were further revealed by bRNA-seq. scRNA-seq was performed to screen hub genes that effectively predict degenerating IVD disease. This study aimed to identify biomarkers that may reveal the mechanism of IDD and elucidate the potential therapeutic target of IDD.

## 2 Materials and methods

### 2.1 IVD tissue collection

Patients who underwent lumbar spine surgery at The Second Affiliated Hospital of Chongqing Medical University provided IVD tissue samples. According to the Pfirrmann classification system for disc degeneration, there was one case of grade I, one case of grade II, three cases of grade Ⅲ and three cases of grade Ⅳ. This study was approved by Ethics Committee of The Second Affiliated Hospital of Chongqing Medical University, and informed consent was also obtained from patients before surgery.

### 2.2 PRO-seq analysis

PRO-seq was performed in human IVD NP tissue. Data acquisition was performed using the SCIEX TripleTOF 5600. *p*-values <0.05 and |log2 fold change) | > 1 were the cutoff criteria. Gene Ontology (GO) and the Kyoto Encyclopedia of Genes and Genomes (KEGG) were performed using OmicsBean (http://www.omicsbean.cn/). Protein-protein interaction (PPI) was conducted in STRING (https://string-db.org/) using Cytoscape software (https://cytoscape.org/). The mass spectrometry proteomics data have been deposited to the ProteomeXchange Consortium *via* the PRIDE (Perez-Riverol et al., 2022) partner repository with the dataset identifier PXD040593.

### 2.3 bRNA-seq analysis

The Gene Expression Omnibus (GEO) database (https://www.ncbi.nlm.nih.gov/gds/) was used to retrieve the bRNA-seq GSE70362 (Kazezian et al., 2015). Differentially expressed genes (DEGs) between grades were identified by analyzing bRNA-seq data with the “limma” package in R software (version 4.2.0). *p*-values <0.05 and |log2 FC| > 0.585 were the cutoff criteria. Machine learning (ML) methods consisted of the random forest (RF) classifier and support vector machine recursive feature elimination (SVM-RFE). The receiver operating characteristic (ROC) curve was analyzed using the R package “pROC".

### 2.4 scRNA-seq analysis

GEO database entries for scRNA-seq GSE199866 (Cherif et al., 2022) and GSE205535 (Li et al., 2022) were downloaded. The R package Seurat (https://satijalab.org/seurat/) was utilized to analyze scRNA-seq data. Using the “tinyarray” (https://github.com/xjsun1221/tinyarray) R package, the expression profiles were compared. The NP cells were annotated according to previews study (Li et al., 2022). The cell-cell communication analysis was conducted using “CellChat” R package.

### 2.5 Animal experiment

Ten male Sprague Dawley rats were obtained from the Animal Experiment Center of Chongqing Medical University. The rats were randomly divided into the normal control group and the degenerative group. In the degenerative group, a 20-gauge micropuncture needle was used to puncture the caudal vertebrae (Co) Co8-9. No treatment was performed in the normal control group. After 4 weeks of treatment, Co8-9 IVDs of the two groups were removed, fixed with 4% paraformaldehyde, and decalcified. The Ethics Committee of The Second Affiliated Hospital of Chongqing Medical University approved all animal experiments.

### 2.6 Hematoxylin and eosin staining

After 4 weeks of decalcification with ethylenediaminetetraacetic acid (EDTA) (Solarbio, China), Co8-9 IVDs were embedded in paraffin. Following the manufacturer’s instructions, paraffin-embedded tissue sections were sliced and stained with hematoxylin and eosin (Beyotime, China).

### 2.7 Immunohistochemical (IHC) staining

Protein expression was detected in different sections using the IHC kit (Zhongshan Jinqiao, China). IHC staining was performed on human and rat IVDs. Paraffin-embedded sections were routinely dewaxed, hydrated, and antigen repaired. The IHC kit was used to detect protein expression following the manufacturer’s protocol. Subsequently, slices were blocked with goat serum and incubated with corresponding primary and secondary antibodies. Sections were stained with diaminobenzidine at room temperature for 40 s and counterstained with hematoxylin for 30 s. IVD tissue slices were dehydrated by ethanol gradients (50, 70, 90, and 100%). Finally, sections were photographed under an upright fluorescence microscope after being sealed with neutral balsam (Solarbio, China).

### 2.8 Gene Set Enrichment Analysis of SERPINC1

Gene Set Enrichment Analysis (GSEA) was performed to show the potential function of SERPINC1. The samples were classified into low- and high-SERPINC1 groups according to the median expression of SERPINC1. The related KEGG dataset was downloaded from MSigDB (http://www.gsea-msigdb.org/gsea/downloads.jsp).

## 3 Results

### 3.1 Identification of differentially expressed proteins (DEPs) in PRO-seq

**FIGURE 1:**
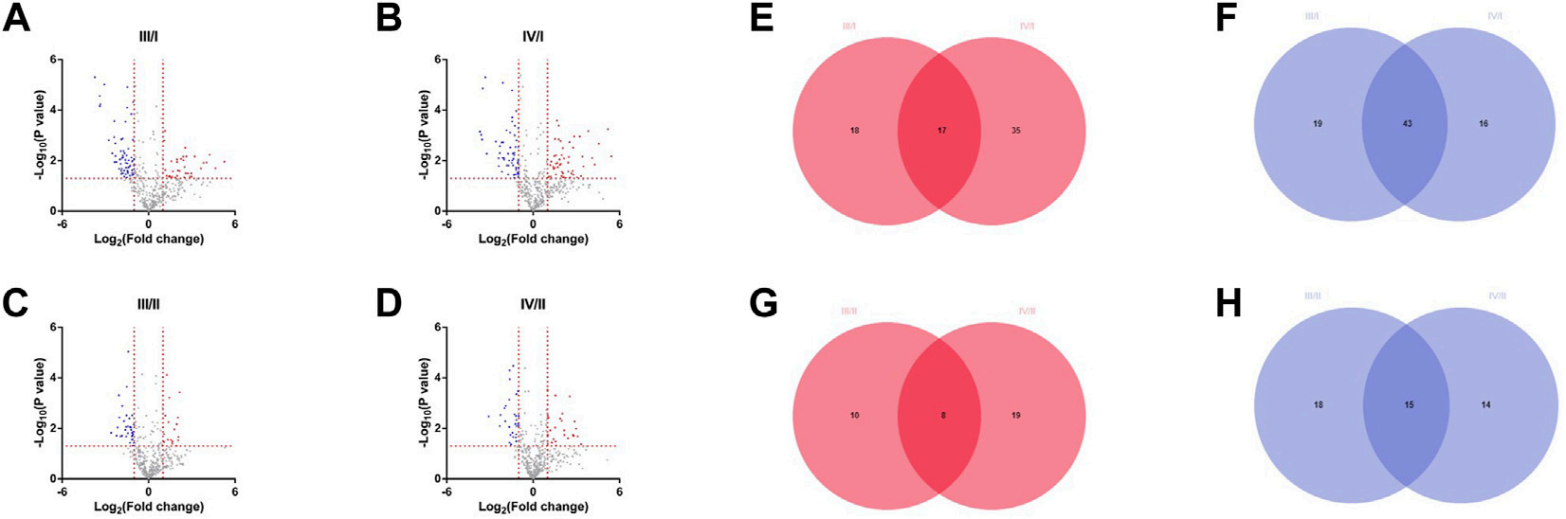
Differentially expressed proteins among different grades. **(A)** The DEPs of grade Ⅲ compared with grade I. **(B)** The DEPs of grade Ⅳ compared with grade I. **(C)** The DEPs of grade Ⅲ compared with grade II. **(D)** The DEPs of grade Ⅳ compared with grade II. **(E)** High expression differential proteins for Ⅲ/I and Ⅳ/I. **(F)** Low expression differential proteins for Ⅲ/I and Ⅳ/I. **(G)** High expression differential proteins for Ⅲ/II and Ⅳ/II. **(H)** Low expression differential proteins for Ⅲ/II and Ⅳ/II.

DEPs between the grades of IVDs were analyzed using PRO-seq data. DEPs were selected based on cutoff criteria (|log2 (fold change| > 1 and *p* < 0.05). There were 35 high-expression and 62 low-expression proteins in grade III/I, 52 high-expression and 59 low-expression proteins in grade IV/I, 18 high-expression and 33 low-expression proteins in grade III/II, and 27 high-expression and 29 low-expression proteins in grade IV/II (Figures 1A–D). Venn diagrams were drawn by taking the intersection of high-expression or low-expression proteins between groups. There were 17 high-expression proteins (Figure 1E) and 43 low-expression proteins (Figure 1F) in the intersection of III/I and IV/I, and 8 high-expression proteins (Figure 1G) and 15 low-expression proteins (Figure 1H) in the intersection of III/II and IV/II, respectively.

### 3.2 Functional enrichment analysis of DEPs in PRO-seq

**FIGURE 2:**
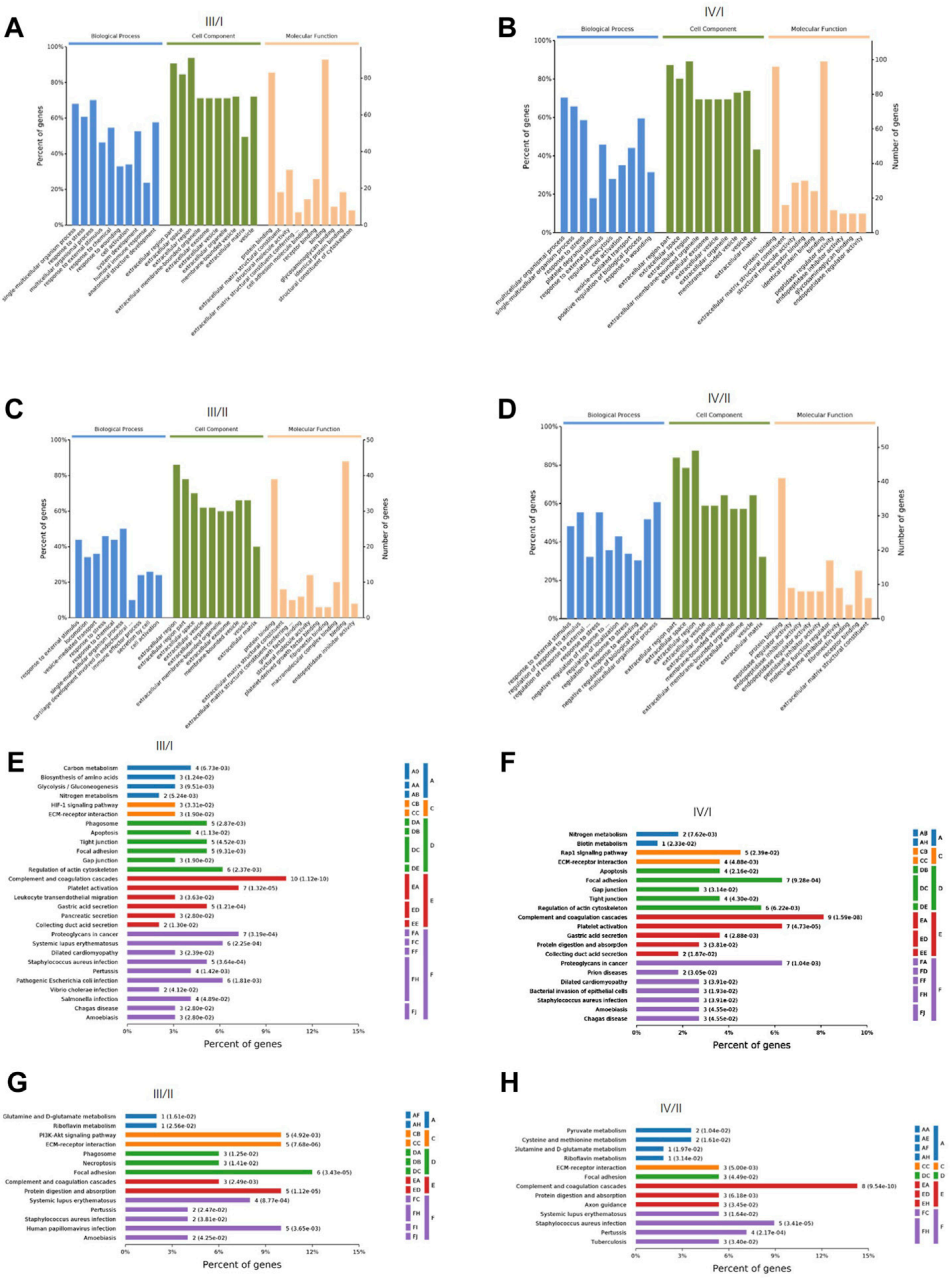
The GO function analysis and KEGG signaling pathway analysis of DEPs. **(A)** GO function analysis for Ⅲ/I. **(B)** GO function analysis for Ⅳ/I. **(C)** GO function analysis for Ⅲ/II. **(D)** GO function analysis for Ⅳ/II. **(E)** KEGG signaling pathway analysis for Ⅲ/I. **(F)** KEGG signaling pathway analysis for Ⅳ/I. **(G)** KEGG signaling pathway analysis for Ⅲ/II. **(H)** KEGG signaling pathway analysis for Ⅳ/II.

GO, and KEGG enrichment analyses of DEPs were performed. The biological process, cell composition, and molecular function of DEPs between III/I, IV/I, III/II, and IV/II are shown in Figures 2A–D. The degree of DEP pathway enrichment between III/I, IV/I, III/II, and IV/II is shown in Figures 2E–H. Functional and pathway enrichment analyses revealed that the protein-encoding genes play multiple functions and involve multiple pathways in the degenerative process of IDD, indicating that IDD is a complex pathophysiological process.

### 3.3 PPI network analysis of DEPs in PRO-seq

**FIGURE 3:**
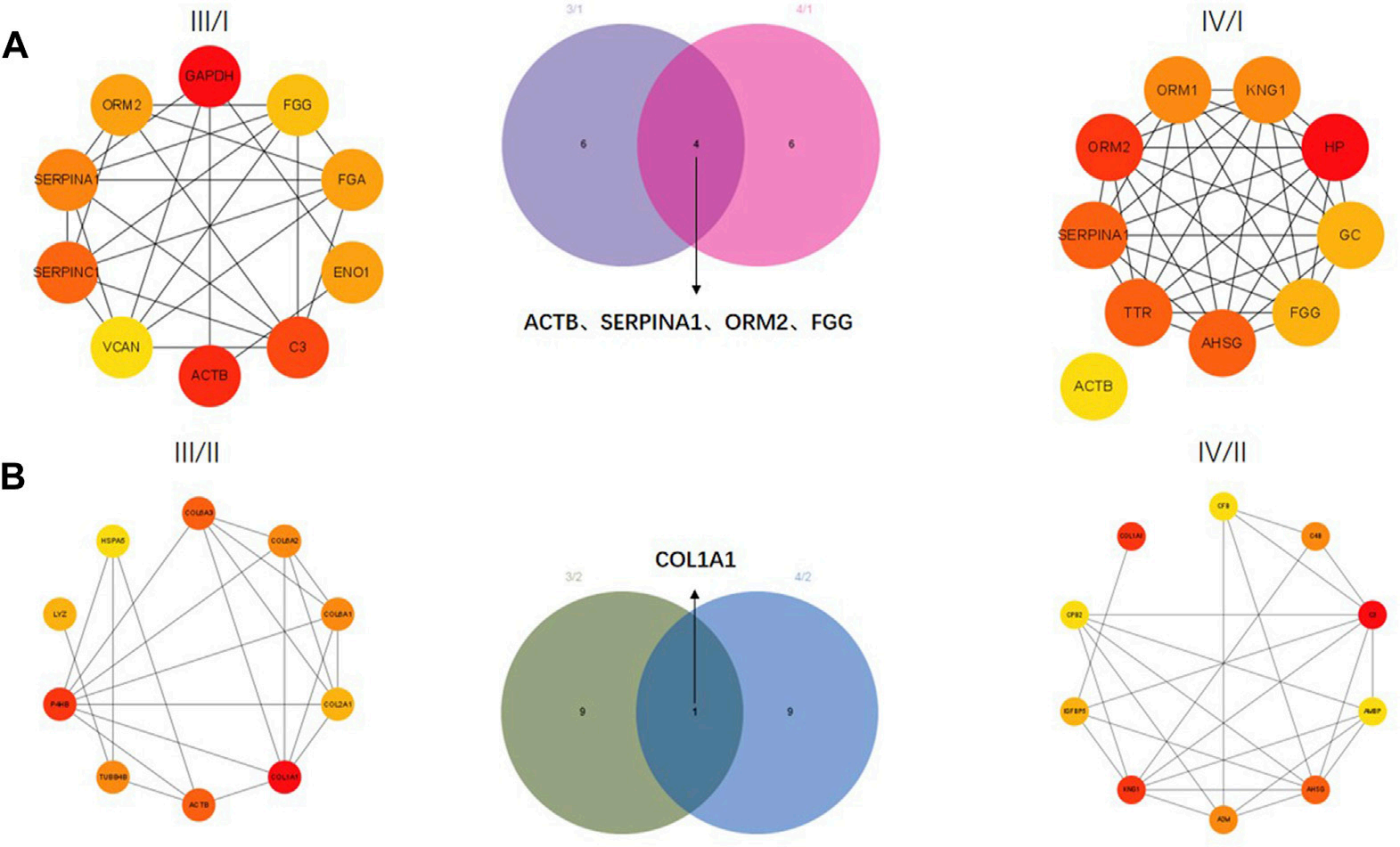
Protein-protein interaction **(**PPI) network analysis of DEPs. **(A)** Core proteins for Ⅲ/I and Ⅳ/I. **(B)** Core proteins for Ⅲ/II and Ⅳ/II.

The correlation between proteins was analyzed by the PPI network. The top 10 core proteins were screened in III/I, IV/I, III/II, and IV/II groups. GAPDH, FGG, FGA, ENO1, C3, ACTB, VCAN, SERPINC1, SERPINA1, and ORM2 were core proteins in the III/I group. ACTB, TTR, AHSG, FGG, GC, HP, KNG1, ORM1, ORM2, and SERPINA1 were core proteins in the IV/I group. ACTB, SERPINA1, ORM2, and FGG were common proteins in the III/I and IV/I groups (Figure 3A). Among them, ACTB was removed due to its expression and interaction. COL6A3, COL6A2, COL6A1, COL2A1, COL1A1, ACTB, TUBB48, P4HB, LYZ, and HSPA5 were core proteins in the III/II group. CFB, C48, C3, AMBP, AHSG, A2M, KNG1, IGFBP5, CPB2, and COL1A1 were core proteins in the IV/II group. COL1A1 was the only common protein in the III/II and IV/II groups (Figure 3B).

### 3.4 DEGs and functional enrichment of bRNA-seq

**FIGURE 4:**
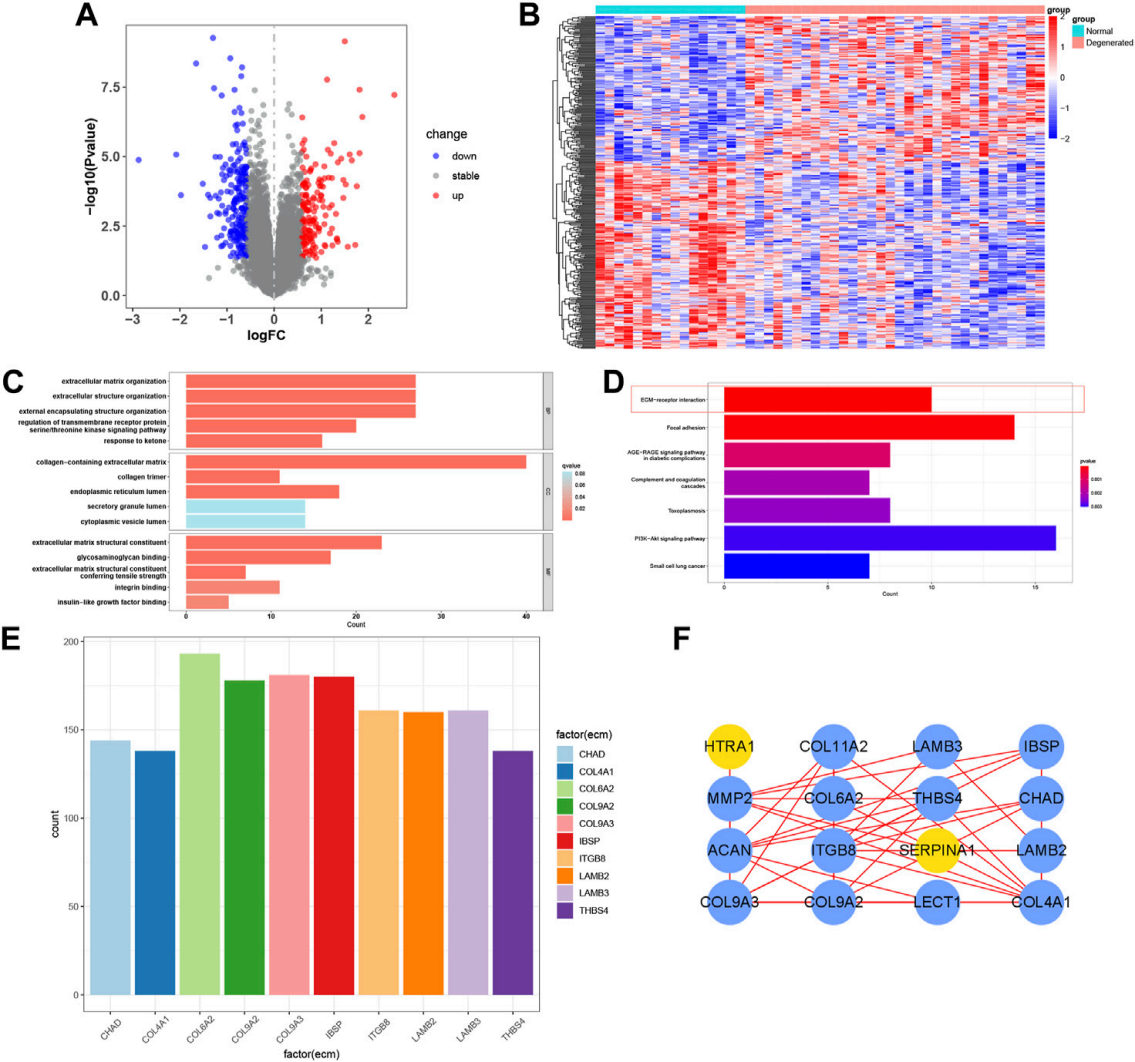
Functional enrichment of bRNA-seq. **(A, B)** Volcano map and heatmap of the two groups in bRNA-seq. **(C, D)** Bar plot of GO and KEGG analysis. **(E)** Correlation maps between DEGs and ECM−receptor interaction factors. **(F)** PPI network of the CGs.

The IDD microarray dataset (GSE70362) was downloaded to explore the hub genes further. The bRNA-seq set was grouped according to the Thompson grading system (Gibson and Waddell, 2007). Grades I and II were considered the normal group, and grades III-V were the degenerated group. A total of 360 DEGs were screened between the two groups (Figures 4A,B). GO and KEGG enrichment analyses were performed to determine the potential pathogenic mechanisms between the two groups (Figures 4C,D). Among them, ECM-receptor interaction, also exhibited in the functional results of PRO-seq, was significant in the degenerative process of IDD (Zhang et al., 2021a). We calculated the correlation between DEGs and ECM-receptor interaction factors with *p* < 0.001 and |Cor-value| > 0.6. Nineteen ECM-receptor interaction-related genes (CGs) were identified (Figure 4E). The PPI network of the CGs is shown in Figure 4F.

### 3.5 Selection of optimal genes for IDD and efficiency validation in bRNA-seq

**FIGURE 5:**
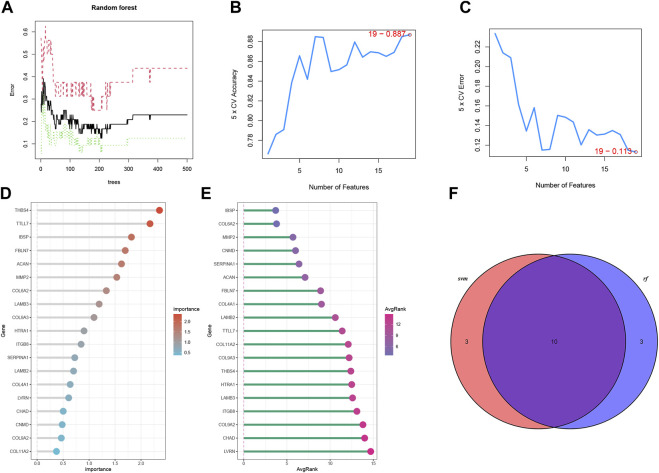
Machine learning algorithms to select optimal genes of IDD in bRNA-seq. **(A)** The features of RF. **(B, C)** The index of SVM-REF. **(D)** Gene importance order of RF. **(E)** Average rank of genes in SVM-REF. **(F)** Venn diagram of the RF and SVM-REF results.

**FIGURE 6:**
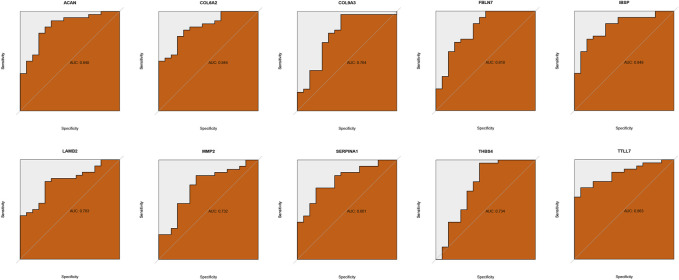
Validation of hub genes predictiveness.

Two ML algorithms (RF and SVM-REF) were used to select candidate genes from CGs to predict IDD. RF and SVM-REF selected 13 genes each (Figures 5A–E). With an intersection of 10 hub genes (IBSP, COL6A2, MMP2, SERPINA1, ACAN, FBLN7, LAMB2, TTLL7, COL9A3, and THBS4), RF and SVM-REF selected optimal hub genes for IDD (Figure 5F). The ROC curve was used to estimate the predictiveness of the hub genes (Figure 6). Among the ten hub genes, four genes had an area under the curve (AUC) > 0.7, indicating predictive performance, and six genes had an AUC >0.8, indicating the highly predictive ability of the hub genes.

### 3.6 scRNA-seq validation for SERPINA1

**FIGURE 7:**
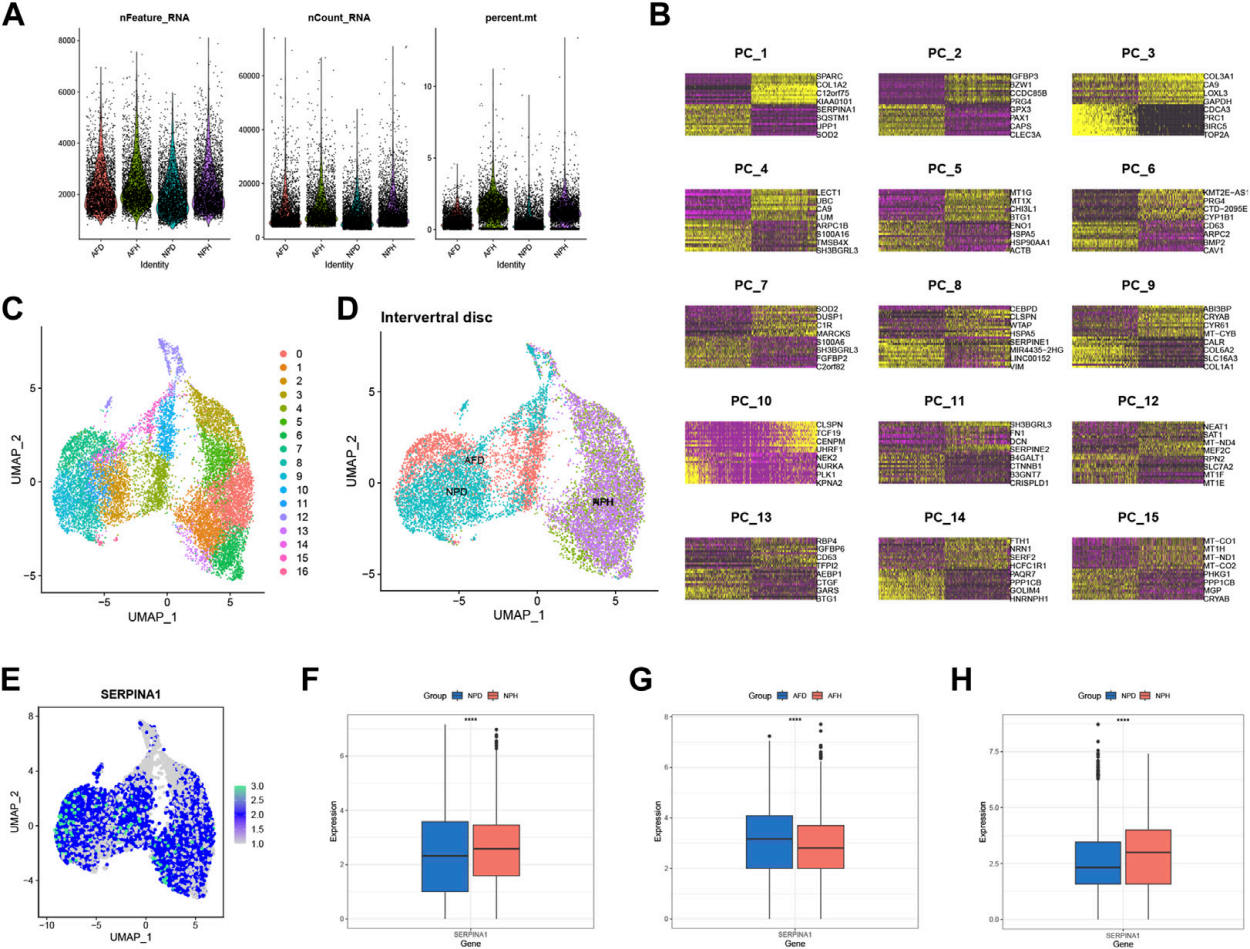
scRNA-seq level validation of SERPINA1. **(A)** Quality-control data of scRNA-seq. **(B)** 1:15 cluster of PCA reduction. **(C)** Dimplot of the 17 clusters after UMP reduction. **(D)** The distribution in Dimplot of AFH, NPH, AFD and NPD. **(E)** The expression and distribution of SERPINA1 in Dimplot. **(F)** The boxplot of SERPINA1′ expression comparison between NPD and NPH. **(G)** The boxplot of SERPINA1′ expression comparison between AFD and AFH. **(H)** The boxplot of SERPINA1′ expression comparison between NPD and NPH in the validation group.

scRNA-seq analysis was performed to validate the expression of SERPINA1. After filtrating the appropriate value of genes (Figure 7A), principal component analysis (PCA) and clustering analysis were performed. Data were divided into several PC clusters. The heatmap showed features of 1:15 PC clusters (Figure 7B). A uniform manifold approximation and projection (UMAP) was conducted for non-linear dimension reduction, and 17 clusters were obtained (Figure 7C). The UMAP plot showed that healthy annulus fibrosus (AFH) and healthy nucleus pulposus (NPH) were relatively overlapping, while degenerated annulus fibrosus (AFD) and degenerated nucleus pulposus (NPD) were almost all overlapped (Figure 7D). The expression of SERPINA1 was highly expressed in a large proportion of cells (Figure 7E); however, the expression was much higher in the NPH compared with the NPD (Figure 7F) but much lower in the AFH compared with the AFD (Figure 7G). An external validation set that only contains NP cells was performed to check SERPINA1 expression in Figure 7H.

### 3.7 Protein expression in human IVDs and rat IVDs

**FIGURE 8:**
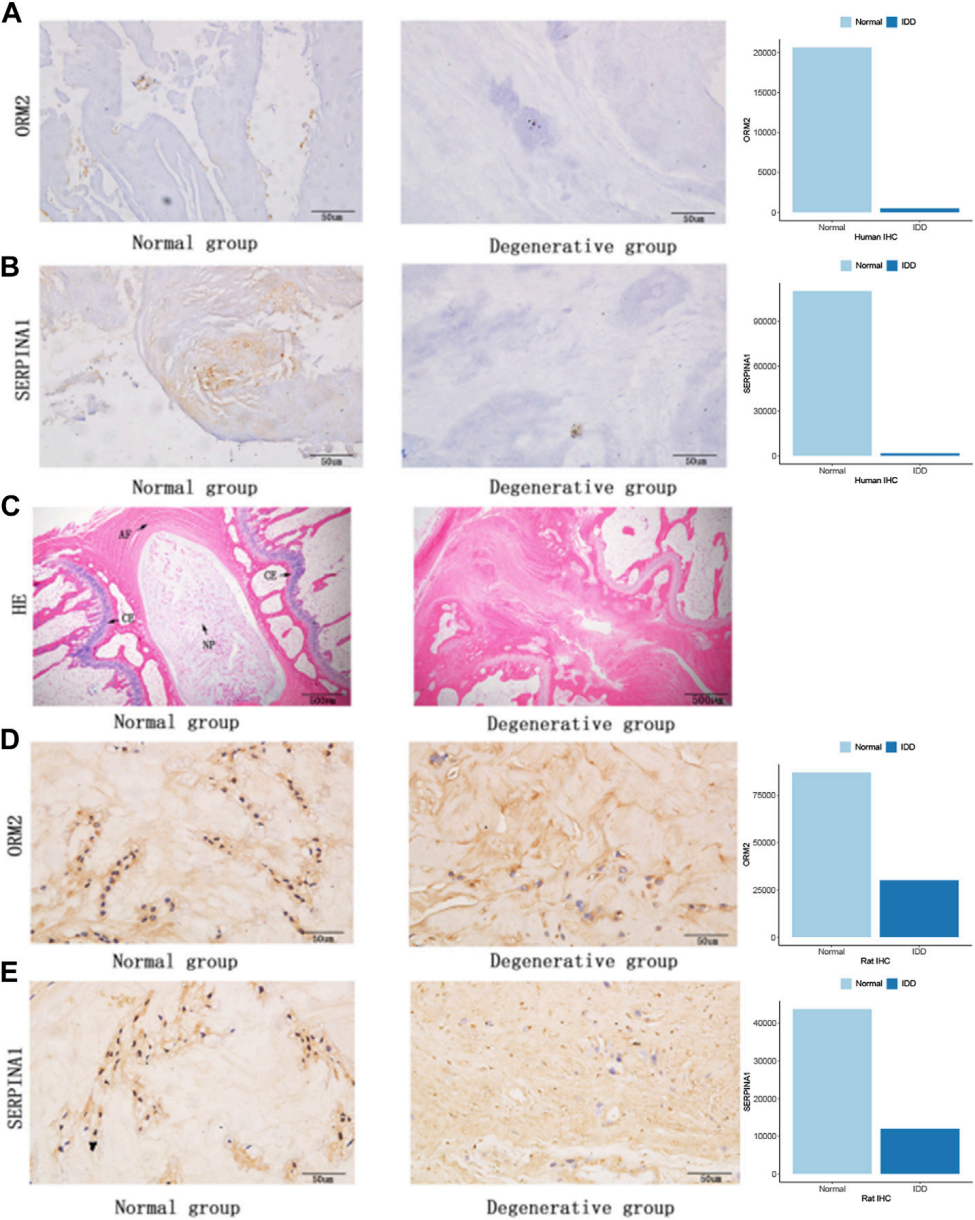
Protein expression in human IVDs and rat IVDs. **(A)** ORM2 expression in human IVDs. **(B)** SERPINA1 expression in human IVDs. **(C)** Hematoxylin and eosin staining of rat IVDs (AF, annulus fibrosus; NP, nucleus pulposus; CE, cartilage endplate). **(D)** ORM2 expression in rat IVDs. **(E)** SERPINA1 expression in rat IVDs.

To further examine the expression of core genes in IVD tissues, we performed IHC staining on human IVD tissues. Therefore, we detected the expression of ORM2 and SERPINA1 proteins in tissues, and the results showed that these expressions were higher in normal tissues than in degenerative tissues (Figures 8A,B). At the same time, we also performed rat animal models of IDD and detected the changes in protein expression in rat IVD tissues. HE staining showed a normal structure of the annulus fibrosus, nucleus pulposus, and cartilage endplate, while the structure of the IVD was disordered in the degenerative group of rats (Figure 8C). We also detected the expression of ORM2 and SERPINA1 proteins in rat IVD tissues. The results showed that the expression of ORM2 and SERPINA1 was higher in normal tissues than in degenerative tissues (Figures 8D,E).

### 3.8 Functions of SERPINA1 in bRNA-seq

**FIGURE 9:**
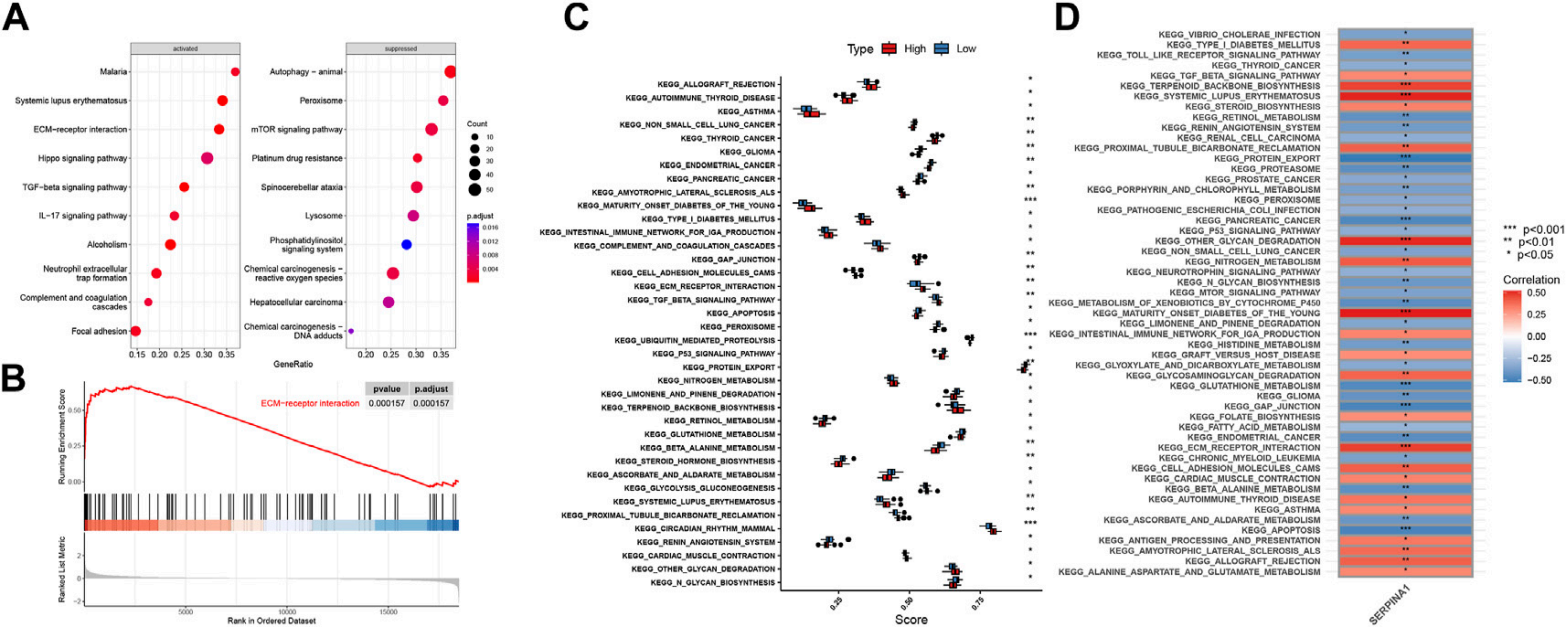
Functions of SERPINA1 in bRNA-seq. **(A)** GSEA results of SERPINA1. **(B)** The enrichment status of SERPINA1 in ECM-receptor interaction pathway. **(C)** Boxplot of pathway enrichment differences in high-SERPINA1 group and low- SERPINA1 group. **(D)** The correlations between SERPINA1 and enriched pathways.

We performed GSEA analysis to explore how SERPINA1 could regulate IDD. SERPINA1 could activate ECM-receptor interaction and suppress peroxisomes (Figure 9A). In addition, the ECM-receptor interaction was more enriched in the high-SERPINA1 group (Figure 9B). In the comparison of the high-SERPINA1 group and low-SERPINA1 group, we found that APOPTOSIS and TGFβ pathways exhibited higher performance in the low-SERPINA1 subtype and high-SERPINA1 respectively (Figure 9C). Furthermore, SERPINA1 correlated significantly positively with MATURITY ONSET DIABETES OF THE YOUNG, but significantly negatively with GAP JUNCTION (Figure 9D).

### 3.9 Functions of SERPINA1 in scRNA-seq

**FIGURE 10:**
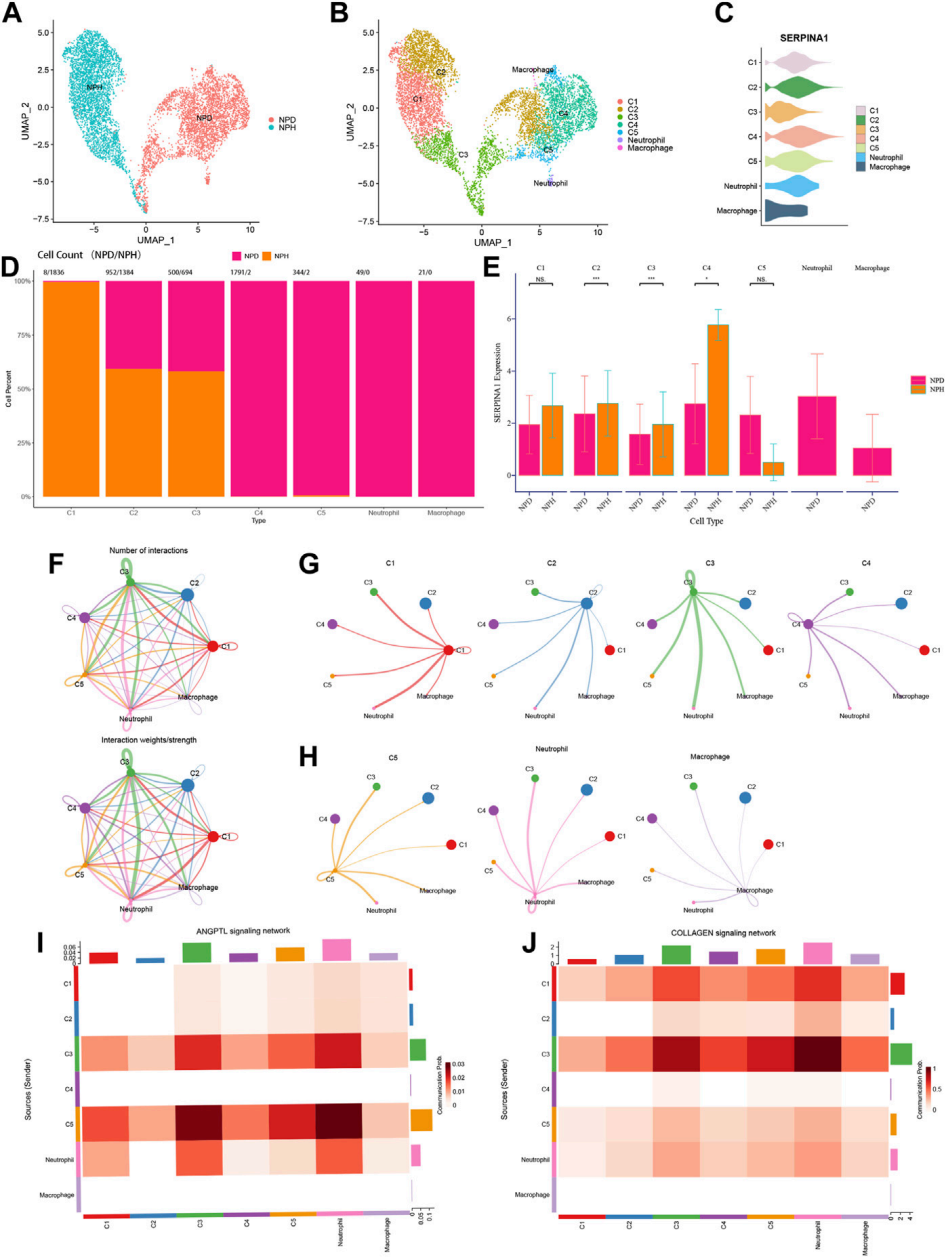
Functions of SERPINA1 in scRNA-seq. **(A)** NPH and NPD cells in scRNA-seq data. **(B)** Dimplot of cell types’ distributions. **(C)** Violin diagram of SERPINA1′ expression in different cell types. **(D)** The percentage of NPD and NPH in each cell type. **(E)** The comparison of SERPINA1′ expression in each cell type. **(F)** The interaction numbers and weights of cell-cell communications. **(G)** Cell-cell communications of C1, C2, C3 and C4. **(H)** Cell-cell communications of C5, neutrophil and macrophage. **(I)** Cell types participate in ANGPTL signaling network. **(J)** Cell types participate in COLLAGEN signaling network.

NP cells of scRNA-seq data were reanalysed to investigate the function of SERPINA1 in different cell types. All NPD and NPH cells were grouped distinctively (Figure 10A). According to the markers, the following types were identified: chondrocytes 1 (C1), chondrocytes 2 (C2), chondrocytes 3 (C3), chondrocytes 4 (C4), chondrocytes 5 (C5), neutrophil, and macrophage (Figure 10B). SERPINA1 expressed sufficiently in C2 and C4 (Figure 10C). C1 was almost exclusively composed of NPH cells, whereas C4 and C5 were almost exclusively composed of NPD cells. And there were no NPH cells in neutrophil and macrophage (Figure 10D). The expressions of SERPINA1 in C1, C2, C3 and C4 were higher in the NPH subtype, and C5 had a lower expression of SERPINA1 in the NPH subtype (Figure 10E). The numbers and weights of cell-cell communications were shown in Figure 10F. In the cell types which expressed SERPINA1 highly in NPH, they had better performance in communicating with other cells (Figure 10G). While the communication intensities of C5, macrophage, and neutrophil were relatively low (Figure 10H). C5 and neutrophil had abundant communication probabilities in ANGPTL signaling network (Figure 10I). The signaling network of COLLAGEN showed better strength in C1 and C3 (Figure 10J).

## 4 Discussion

IDD is the most common cause of LBP (Wang et al., 2020). Pathophysiological changes associated with IDD are complex, and its degeneration mechanism is not entirely understood. IVD degeneration is an irreversible condition frequently linked to aging (Dowdell et al., 2017). Its clinical treatment mainly includes anti-inflammatory treatment and surgical resection or replacement of intervertebral discs (Mirza and Deyo, 2007). Nevertheless, these methods are only symptomatic treatments, and it is difficult to reverse the degenerative disc. The surgical treatment of intervertebral discs also burdens patients economically (Fiordalisi et al., 2021). Therefore, exploring the core genes and pathogenesis of intervertebral discs is essential, which may provide experimental and theoretical bases for the repair of IDD. In the present study, we performed multi-omics sequencing analyses in different IDD grades to identify the key hub genes and evaluate the function of the core hub genes. Five genes were identified (ACTB, COL1A1, SERPINA1, ORM2, and FGG) *via* PRO-seq by analyzing DEPs in different grades of human IVDs. Actin beta (ACTB) is a housekeeping gene and one of the cytoskeletal actins (Cuvertino et al., 2017). By analyzing data from gene microarrays (GSE23130) and microRNA expression microarrays (GSE63492), Mo et al. found that ACTB was highly expressed in degenerative tissues (Mo et al., 2019). Cui et al. found that ACTB was highly expressed in degeneration through sequencing the transcriptome of IVDs after one strike loading on the simulated disc degeneration of bovine (Cui et al., 2021). Actin gamma 1 (ACTG1), a member of the actin family, negatively correlates with the grades of human IDD (Wu et al., 2021a). F-actin cytoskeletal may also play a regulatory role in disc degeneration (Li et al., 2007). Cytoskeletal actin changes may affect the communication between cells and the extracellular matrix. During degeneration, the extracellular matrix of the intervertebral disc undergoes alterations, which are primarily manifested by the decrease in polysaccharide and collagen synthesis and the increase in decomposition (Sakai and Grad, 2015). The extracellular matrix of IVD is composed mainly of collagen, with type I collagen representing the primary constituent of the annulus fibrosus (Yang et al., 2020) and type II collagen serving as the main component secreted by nucleus pulposus cells (Fernandes et al., 2020). Correlative research demonstrated an association between collagen gene polymorphisms and IVD deterioration (Trefilova et al., 2021). Polymorphisms in COL1A1 Sp1 and COL11A1 C4603T are associated with IDD risk, and these collagen genes may be used to treat or prevent IDD (Xie et al., 2021). Fibrinogen, a non-collagen glycoprotein, is an important part of the extracellular matrix (Schuppan et al., 1998). The fibrinogen gamma chain (FGG) peptide was coated with adenosine diphosphate encapsulated liposomes, which played a hemostatic role in thrombocytopenic rabbits and caused lethal blast lung injury in mice (Hagisawa et al., 2016; Hagisawa et al., 2019). We found that the expression of FGG varied between degenerative and normal tissues. It may be used as a hub gene to regulate the IDD process. However, the effect of FGG on degenerative IVDs has not been observed, and further research is required to determine its role and specific mechanism in IVDs.

Further data exploration was conducted with bRNA-seq, and the functional enrichment analysis was performed based on the DEGs. GO analysis showed that collagen-containing extracellular matrix and glycosaminoglycan binding were enriched significantly. As a key structure in both the AF and NP of IVDs, changes in collagen content with degeneration indicate shifts from collagen type II to type I within the NP (Zeldin et al., 2020). Negatively charged aggrecan glycosaminoglycans can provide compressive stiffness, hydration, and swelling pressures to tissue, which leads to a high negative fixed charge density in NP (Wagner et al., 2020). KEGG analysis showed that the PI3K−Akt signaling pathway and ECM-receptor interaction were enriched. We focused on the ECM-receptor interaction since ECM degradation is one of the leading causes of IDD (Sun et al., 2021). ML algorithms can be applied to analyze complex medical data and may guide clinical and nursing practice (Deo, 2015). Using ML methods, ten genes were selected, including IBSP, COL6A2, MMP2, SERPINA1, ACAN, FBLN7, LAMB2, TTLL7, COL9A3, and THBS4. COL6A2 is one type of collagen VI gene, a crucial component of the extracellular matrix. It forms a microfibrillar network closely associated with the cell and surrounding basement membrane (Bushby et al., 2014). It was reported that in the absence of the interleukin 1 beta T allele, the carriage of COL9A3 (Trp3 allele) increased the risk of dark nucleus pulposus (Solovieva et al., 2006). SERPINA1 was identified in both PRO-seq and bRNA-seq. We also explored the potential function of SERPINA1 in the bRNA-seq, with the expression variation of SERPINA1, the enrichments of the APOPTOSIS pathway, TGFβ pathway, MATURITY ONSET DIABETES OF THE YOUNG pathway, and GAP JUNCTION pathway change as well. These pathways have already been proven to work in the process of IDD (Gruber et al., 2001; Cazzanelli and Wuertz-Kozak, 2020; Wu et al., 2021b). scRNA-seq has an advantage over bRNA-seq because it has an extracellular dimension. Further validation was thus performed in scRNA-seq, and the results showed that the expression of SERPINA1 was much higher in the normal group of NP tissue. In the different types of chondrocytes, C1, C2, C3, and C4 had higher SERPINA1 expressions in NPH compared with NPD, and they had better cell-cell communication intensities. But in C5, the expression of SERPINA1 was inverse, and the communication stayed in a low strength. Chondrocyte 4 (C4) is an inflamed chondrocyte (Li et al., 2022), and SERPINA1 expressed abundantly in C4. Although there were few NPH cells in C4, the expression of SERPINA1 was much greater in NPH compared with NPD. The underlying mechanisms are meaningful and worthy for our further exploring. Angiopoietin-like protein (ANGPTL) is structurally similar to angiopoietin, which facilitates the process of angiogenesis (Neto et al., 2019). And angiogenesis factor is involved in the development of IDD (David et al., 2010). With a high SERPINA1 expression in NPD, C5 performed well in the ANGPTL signaling network. Collagen is a part of NP, and its dysfunction occurs with IVD degeneration (Trefilova et al., 2021). C1 and C3 had higher SERPINA1 expressions in NPH, and their communications in COLLAGEN signaling network exhibited higher strength. Therefore, SERPINA1 was a pivotal gene for IDD.

Based on three structures, the nucleus pulposus, annulus fibrosus, and cartilage endplate in the normal intervertebral disc (Yang et al., 2020), we used a micropuncture needle to puncture the Co8-9 intervertebral disc of rats. The degenerative tissue contained a few nucleus pulposus cells, a damaged annulus fibrosus, and degenerative cartilage endplates (Figure 8C). Degeneration destroys the structure of IVDs, affecting the internal balance of the disc. These results demonstrated that the model of degeneration in the caudal vertebra of rats was successfully established. We verified the expression of SERPINA1 in human and rat intervertebral discs using IHC staining. The expressions of SERPINA1 were lower in human and rat degenerating tissues than in normal tissues. SERPINA1 is a plasma protein and a serine protease inhibitor mainly expressed in the liver (Niemietz et al., 2020). A previous study showed that SERPINA1 peptides in urine could be used as potential markers for diagnosing the severity of preeclampsia (Starodubtseva et al., 2020). We also attempted to verify the expression of other hub genes in human and rat intervertebral discs. Interestingly, orosomucoid 2 (ORM2) expression was significantly higher in the normal NP compared with the degenerated counterpart. ORM (an acute phase protein) is a group of small molecule-binding proteins with immunomodulatory function (Jo et al., 2017). According to Zhu et al., high ORM2 expression was associated with a better prognosis in liver cancer patients (Zhu et al., 2020). However, there are few studies on ORM2 and SERPINA1 in IVDs.

In summary, we conclude that SERPINA1 is decreased in the NP tissue of IDD based on multi-omics analyses and experimental findings, suggesting that it could serve as a hub gene and a potential treatment target to regulate the process of IDD. The related pathway mechanism and target genes merit further investigation. This may provide a theoretical basis for reversing or repairing IDD in the future.

## Data Availability

The data presented in the study are deposited in the ProteomeXchange Consortium, accession number PXD040593.

## References

[B1] Borenstein D. Balagué F. (2021). Low back pain in adolescent and geriatric populations. Rheumatic Dis. Clin. N. Am. 47 (2), 149–163. 10.1016/j.rdc.2020.12.001 33781487

[B2] Bushby K. Collins J. Hicks D. (2014). Collagen type VI myopathies. Adv. Exp. Med. Biol. 802, 185–199. 10.1007/978-94-007-7893-1_12 24443028

[B3] Cazzanelli P. Wuertz-Kozak K. (2020). MicroRNAs in intervertebral disc degeneration, apoptosis, inflammation, and mechanobiology. Int. J. Mol. Sci. 21 (10), 3601. 10.3390/ijms21103601 32443722 PMC7279351

[B4] Chao-Yang G. Peng C. Hai-Hong Z. (2021). Roles of NLRP3 inflammasome in intervertebral disc degeneration. Osteoarthr. Cartil. 29 (6), 793–801. 10.1016/j.joca.2021.02.204 33609693

[B5] Che H. Li J. Li Y. Ma C. Liu H. Qin J. (2020). p16 deficiency attenuates intervertebral disc degeneration by adjusting oxidative stress and nucleus pulposus cell cycle. Elife 9, e52570. 10.7554/eLife.52570 32125276 PMC7065909

[B6] Cheng X. Zhang L. Zhang K. Zhang G. Hu Y. Sun X. (2018). Circular RNA VMA21 protects against intervertebral disc degeneration through targeting miR-200c and X linked inhibitor-of-apoptosis protein. Ann. Rheum. Dis. 77 (5), 770–779. 10.1136/annrheumdis-2017-212056 29343508 PMC5909753

[B7] Cherif H. Mannarino M. Pacis A. Ragoussis J. Rabau O. Ouellet J. (2022). Single-cell RNA-seq analysis of cells from degenerating and non-degenerating intervertebral discs from the same individual reveals new biomarkers for intervertebral disc degeneration. Int. J. Mol. Sci. 23 (7), 3993. 10.3390/ijms23073993 35409356 PMC8999935

[B8] Cui S. Zhou Z. Chen X. Wei F. Richards R. Alini M. (2021). Transcriptional profiling of intervertebral disc in a post-traumatic early degeneration organ culture model. JOR spine 4 (3), e1146. 10.1002/jsp2.1146 34611583 PMC8479529

[B9] Cuvertino S. Stuart H. Chandler K. Roberts N. Armstrong R. Bernardini L. (2017). ACTB loss-of-function mutations result in a pleiotropic developmental disorder. Am. J. Hum. Genet. 101 (6), 1021–1033. 10.1016/j.ajhg.2017.11.006 29220674 PMC5812896

[B10] David G. Ciurea A. V. Iencean S. M. Mohan A. (2010). Angiogenesis in the degeneration of the lumbar intervertebral disc. J. Med. Life 3 (2), 154–161. 20968201 PMC3019053

[B11] Deo R. C. (2015). Machine learning in medicine. Circulation 132 (20), 1920–1930. 10.1161/CIRCULATIONAHA.115.001593 26572668 PMC5831252

[B12] Dowdell J. Erwin M. Choma T. Vaccaro A. Iatridis J. Cho S. (2017). Intervertebral disk degeneration and repair. Neurosurgery 80, S46–S54. 10.1093/neuros/nyw078 28350945 PMC5585783

[B13] Fernandes L. Khan N. Trochez C. Duan M. Diaz-Hernandez M. Presciutti S. (2020). Single-cell RNA-seq identifies unique transcriptional landscapes of human nucleus pulposus and annulus fibrosus cells. Sci. Rep. 10 (1), 15263. 10.1038/s41598-020-72261-7 32943704 PMC7499307

[B14] Fiordalisi M. Silva A. Barbosa M. Gonçalves R. Caldeira J. (2021). Intervertebral disc decellularisation: Progress and challenges. Eur. cells Mater. 42, 196–219. 10.22203/eCM.v042a15 34613611

[B15] Gibson J. Waddell G. (2007). Surgical interventions for lumbar disc prolapse: Updated cochrane review. Spine 32 (16), 1735–1747. 10.1097/BRS.0b013e3180bc2431 17632394

[B16] Gruber H. E. Ma D. Hanley E. N. Jr. Ingram J. Yamaguchi D. T. (2001). Morphologic and molecular evidence for gap junctions and connexin 43 and 45 expression in annulus fibrosus cells from the human intervertebral disc. J. Orthop. Res. 19 (5), 985–989. 10.1016/S0736-0266(00)00072-3 11562151

[B17] Hagisawa K. Kinoshita M. Miyawaki H. Sato S. Miyazaki H. Takeoka S. (2016). Fibrinogen γ-chain peptide-coated adenosine 5' diphosphate-encapsulated liposomes rescue mice from lethal blast lung injury via adenosine signaling. Crit. care Med. 44 (9), e827–e837. 10.1097/ccm.0000000000001707 27054893

[B18] Hagisawa K. Kinoshita M. Takikawa M. Takeoka S. Saitoh D. Seki S. (2019). Combination therapy using fibrinogen γ-chain peptide-coated, ADP-encapsulated liposomes and hemoglobin vesicles for trauma-induced massive hemorrhage in thrombocytopenic rabbits. Transfusion 59 (10), 3186–3196. 10.1111/trf.15427 31257633

[B19] Hartvigsen J. Hancock M. Kongsted A. Louw Q. Ferreira M. Genevay S. (2018). What low back pain is and why we need to pay attention. Lancet (London, Engl. 391 (10137), 2356–2367. 10.1016/s0140-6736(18)30480-x 29573870

[B20] Hickman T. Rathan-Kumar S. Peck S. (2022). Development, pathogenesis, and regeneration of the intervertebral disc: Current and future insights spanning traditional to omics methods. Front. Cell Dev. Biol. 10, 841831. 10.3389/fcell.2022.841831 35359439 PMC8963184

[B21] Hoy D. Bain C. Williams G. March L. Brooks P. Blyth F. (2012). A systematic review of the global prevalence of low back pain. Arthritis rheumatism 64 (6), 2028–2037. 10.1002/art.34347 22231424

[B22] Jo M. Kim J. Song G. Seo M. Hwang E. Suk K. (2017). Astrocytic orosomucoid-2 modulates microglial activation and neuroinflammation. J. Soc. Neurosci. 37 (11), 2878–2894. 10.1523/jneurosci.2534-16.2017 PMC6596722 28193696

[B23] Kazezian Z. Gawri R. Haglund L. Ouellet J. Mwale F. Tarrant F. (2015). Gene expression profiling identifies interferon signalling molecules and IGFBP3 in human degenerative annulus fibrosus. Sci. Rep. 5, 15662. 10.1038/srep15662 26489762 PMC4614807

[B24] Li S. Duance V. Blain E. (2007). F-actin cytoskeletal organization in intervertebral disc health and disease. Biochem. Soc. Trans. 35, 683–685. 10.1042/bst0350683 17635121

[B25] Li Y. Pan D. Liu S. Xing X. Zhou H. Zhang B. (2021). Identification of circ-FAM169A sponges miR-583 involved in the regulation of intervertebral disc degeneration. J. Orthop. Transl. 26, 121–131. 10.1016/j.jot.2020.07.007 PMC7773979 33437631

[B26] Li Z. Ye D. Dai L. Xu Y. Wu H. Luo W. (2022). Single-cell RNA sequencing reveals the difference in human normal and degenerative nucleus pulposus tissue profiles and cellular interactions. Front. Cell Dev. Biol. 10, 910626. 10.3389/fcell.2022.910626 35874809 PMC9301035

[B27] Mirza S. Deyo R. (2007). Systematic review of randomized trials comparing lumbar fusion surgery to nonoperative care for treatment of chronic back pain. Spine 32 (7), 816–823. 10.1097/01.brs.0000259225.37454.38 17414918

[B28] Mo S. Liu C. Chen L. Ma Y. Liang T. Xue J. (2019). KEGG-expressed genes and pathways in intervertebral disc degeneration: Protocol for a systematic review and data mining. Medicine 98 (21), e15796. 10.1097/md.0000000000015796 31124977 PMC6571259

[B29] Morris C. Cluet D. Ricci E. (2021). Ribosome dynamics and mRNA turnover, a complex relationship under constant cellular scrutiny. Wiley Interdiscip. Rev. RNA 12 (6), e1658. 10.1002/wrna.1658 33949788 PMC8519046

[B30] Neto N. I. P. Boldarine V. T. Hachul A. C. L. Oyama L. M. Lima J. Fernandez E. S. (2019). Association between ANGPTL-4 and the proinflammatory process in cancer cachexia patients. Oncotarget 10 (60), 6444–6455. 10.18632/oncotarget.27269 31741709 PMC6849656

[B31] Niemietz C. Bezerra F. Almeida M. Guo S. Monia B. Saraiva M. (2020). SERPINA1 modulates expression of amyloidogenic transthyretin. Exp. Cell Res. 395 (2), 112217. 10.1016/j.yexcr.2020.112217 32768500

[B32] Perez-Riverol Y. Bai J. Bandla C. Garcia-Seisdedos D. Hewapathirana S. Kamatchinathan S. (2022). The PRIDE database resources in 2022: A hub for mass spectrometry-based proteomics evidences. Nucleic Acids Res. 50 (1), D543–D552. 10.1093/nar/gkab1038 34723319 PMC8728295

[B33] Pfirrmann C. Metzdorf A. Zanetti M. Hodler J. Boos N. (2001). Magnetic resonance classification of lumbar intervertebral disc degeneration. Spine 26 (17), 1873–1878. 10.1097/00007632-200109010-00011 11568697

[B34] Roughley P. Martens D. Rantakokko J. Alini M. Mwale F. Antoniou J. (2006). The involvement of aggrecan polymorphism in degeneration of human intervertebral disc and articular cartilage. Eur. cells Mater. 11, 1–7. 10.22203/ecm.v010a01 16425147

[B35] Sakai D. Grad S. (2015). Advancing the cellular and molecular therapy for intervertebral disc disease. Adv. drug Deliv. Rev. 84, 159–171. 10.1016/j.addr.2014.06.009 24993611

[B36] Schuppan D. Schmid M. Somasundaram R. Ackermann R. Ruehl M. Nakamura T. (1998). Collagens in the liver extracellular matrix bind hepatocyte growth factor. Gastroenterology 114 (1), 139–152. 10.1016/s0016-5085(98)70642-0 9428228

[B37] Solovieva S. Lohiniva J. Leino-Arjas P. Raininko R. Luoma K. Ala-Kokko L. (2006). Intervertebral disc degeneration in relation to the COL9A3 and the IL-1ss gene polymorphisms. Eur. spine J. 15 (5), 613–619. 10.1007/s00586-005-0988-1 16133074 PMC3489335

[B38] Starodubtseva N. Nizyaeva N. Baev O. Bugrova A. Gapaeva M. Muminova K. (2020). SERPINA1 peptides in urine as A potential marker of preeclampsia severity. Int. J. Mol. Sci. 21 (3), 914. 10.3390/ijms21030914 32019243 PMC7037458

[B39] Sun K. Jing X. Guo J. Yao X. Guo F. (2021). Mitophagy in degenerative joint diseases. Autophagy 17 (9), 2082–2092. 10.1080/15548627.2020.1822097 32967533 PMC8496714

[B40] Trefilova V. V. Shnayder N. A. Petrova M. M. Kaskaeva D. S. Tutynina O. V. Petrov K. V. (2021). The role of polymorphisms in collagen-encoding genes in intervertebral disc degeneration. Biomolecules 11 (9), 1279. 10.3390/biom11091279 34572492 PMC8465916

[B41] Wagner E. K. Vedadghavami A. Jacobsen T. D. Goel S. A. Chahine N. O. Bajpayee A. G. (2020). Avidin grafted dextran nanostructure enables a month-long intra-discal retention. Sci. Rep. 10 (1), 12017. 10.1038/s41598-020-68351-1 32694557 PMC7374582

[B42] Wang W. Yu X. Wang C. Yang W. He W. Zhang S. (2015). MMPs and ADAMTSs in intervertebral disc degeneration. Clin. chimica acta; Int. J. Clin. Chem. 448, 238–246. 10.1016/j.cca.2015.06.023 26162271

[B43] Wang Y. Che M. Xin J. Zheng Z. Li J. Zhang S. (2020). The role of IL-1β and TNF-α in intervertebral disc degeneration. Biomed. Pharmacother. = Biomedecine Pharmacother. 131, 110660. 10.1016/j.biopha.2020.110660 32853910

[B44] Wu T. Jia X. Feng H. Wu D. (2021a). ACTG1 regulates intervertebral disc degeneration via the NF-κB-p65 and Akt pathways. Biochem. biophysical Res. Commun. 545, 54–61. 10.1016/j.bbrc.2021.01.057 33545632

[B45] Wu T. Li X. Jia X. Zhu Z. Lu J. Feng H. (2021b). Kruppel like factor 10 prevents intervertebral disc degeneration via TGF-beta signaling pathway both *in vitro* and *in vivo* . J. Orthop. Transl. 29, 19–29. 10.1016/j.jot.2021.04.003 PMC8141503 34094855

[B46] Xie G. Liang C. Yu H. Zhang Q. (2021). Association between polymorphisms of collagen genes and susceptibility to intervertebral disc degeneration: A meta-analysis. J. Orthop. Surg. Res. 16 (1), 616. 10.1186/s13018-021-02724-8 34663366 PMC8522091

[B47] Yang S. Zhang F. Ma J. Ding W. (2020). Intervertebral disc ageing and degeneration: The antiapoptotic effect of oestrogen. Ageing Res. Rev. 57, 100978. 10.1016/j.arr.2019.100978 31669486

[B48] Zeldin L. Mosley G. E. Laudier D. Gallate Z. S. Gansau J. Hoy R. C. (2020). Spatial mapping of collagen content and structure in human intervertebral disk degeneration. JOR Spine 3 (4), e1129. 10.1002/jsp2.1129 33392461 PMC7770200

[B49] Zhang G. Han S. Kong M. Tu Q. Zhang L. Ma X. (2021b). Single-cell RNA-seq analysis identifies unique chondrocyte subsets and reveals involvement of ferroptosis in human intervertebral disc degeneration. Osteoarthr. Cartil. 29 (9), 1324–1334. 10.1016/j.joca.2021.06.010 34242803

[B50] Zhang G. Liu M. Chen H. Wu Z. Gao Y. Ma Z. (2021a). NF-κB signalling pathways in nucleus pulposus cell function and intervertebral disc degeneration. Cell Prolif. 54 (7), e13057. 10.1111/cpr.13057 34028920 PMC8249791

[B51] Zhu H. Zhou W. Wan Y. Ge K. Lu J. Jia C. (2020). Downregulation of orosomucoid 2 acts as a prognostic factor associated with cancer-promoting pathways in liver cancer. World J. gastroenterology 26 (8), 804–817. 10.3748/wjg.v26.i8.804 PMC7052533 32148378

